# Protein structure prediction by *AlphaFold*2: are attention and symmetries all you need?

**DOI:** 10.1107/S2059798321007531

**Published:** 2021-07-29

**Authors:** Nazim Bouatta, Peter Sorger, Mohammed AlQuraishi

**Affiliations:** a Laboratory of Systems Pharmacology, Harvard Medical School, Boston, MA 02115, USA; b Department of Systems Biology, Columbia University, New York, NY 10032, USA

**Keywords:** *AlphaFold*2, protein structure prediction, CASP14

## Abstract

The functions of most proteins result from their 3D structures, but determining their structures experimentally remains a challenge, despite steady advances in crystallography, NMR and single-particle cryoEM. Computationally predicting the structure of a protein from its primary sequence has long been a grand challenge in bioinformatics, intimately connected with understanding protein chemistry and dynamics. Recent advances in deep learning, combined with the availability of genomic data for inferring co-evolutionary patterns, provide a new approach to protein structure prediction that is complementary to longstanding physics-based approaches. The outstanding performance of *AlphaFold*2 in the recent Critical Assessment of protein Structure Prediction (CASP14) experiment demonstrates the remarkable power of deep learning in structure prediction. In this perspective, we focus on the key features of *AlphaFold*2, including its use of (i) attention mechanisms and Transformers to capture long-range dependencies, (ii) symmetry principles to facilitate reasoning over protein structures in three dimensions and (iii) end-to-end differentiability as a unifying framework for learning from protein data. The rules of protein folding are ultimately encoded in the physical principles that underpin it; to conclude, the implications of having a powerful computational model for structure prediction that does not explicitly rely on those principles are discussed.

## Abstract 2

This review discusses the *AlphaFold*2 system for protein structure prediction, including its conceptual and methodological advances, its amenability to interpretation and its achievements in the last Critical Assessment of protein Structure Prediction (CASP14) experiment.

## 1. Introduction   

Determining the 3D structure of a protein from knowledge of its primary (amino-acid) sequence has been a fundamental problem in structural biology since Anfinsen’s classic 1961 refolding experiment, in which it was shown that the folded structure of a protein is encoded in its amino-acid sequence (with important exceptions; Anfinsen *et al.*, 1961 ▸). Last December, the organizers of the Fourteenth Critical Assessment of Structure Prediction (CASP14) experiment made the surprising announcement that DeepMind, the London-based and Google-owned artificial intelligence research group, had ‘solved’ the protein-folding problem (The AlphaFold Team, 2020 ▸) using their *AlphaFold*2 algorithm (Jumper *et al.*, 2021 ▸). Venki Ramakrishnan, past president of the Royal Society and 2009 Nobel Laureate, concluded that ‘[DeepMind’s] work represents a stunning advance on the protein-folding problem, a 50-year-old grand challenge in biology’ (The AlphaFold Team, 2020 ▸). As the initial excitement subsides, it is worth revisiting what it means to have ‘solved’ protein folding and the extent to which *AlphaFold*2 has advanced our understanding of the physical and chemical principles that govern it.

Predicting the fold of a protein from its primary sequence represents a series of related problems (Dill & MacCallum, 2012 ▸; Dill *et al.*, 2008 ▸). One involves elucidating the physical principles and dynamical processes underlying the conversion of a newly synthesized protein chain into a three-dimensional structure. Given a sufficiently accurate energy model, for example a general solution of the all-atom Schrödinger equation, solving protein folding reduces to simulating dynamical equations of the motion of polypeptides in solution until the lowest free-energy state is reached. Unfortunately, this calculation is impossible given contemporary computers. In modern practical simulations, interatomic interactions are described by approximate energy models, and folding dynamics are captured by iteratively solving Newton’s dynamical laws of motion (mostly ignoring quantum effects; Karplus & McCammon, 2002 ▸; Karplus & Petsko, 1990 ▸). This is the purview of molecular dynamics, and despite impressive advances, including the development of specialized computing hardware (Grossman *et al.*, 2015 ▸), *de novo* folding of proteins using molecular dynamics remains limited to small proteins ranging from ten to 80 amino-acid residues (Shaw *et al.*, 2010 ▸; Lindorff-Larsen *et al.*, 2011 ▸).

A second question, more aptly termed a paradox, was first raised in a *gedankenexperiment* by Levinthal (1968 ▸). While relaxing from an unfolded to a native state, a protein has the potential to explore a dauntingly large number of conformations. If this process were random and unbiased, as was originally assumed, a 100-residue protein would take ∼10^52^ years to fold, longer than the current age of the universe (Karplus, 1997 ▸). In practice, most single-domain proteins fold in milliseconds to seconds. To address this discrepancy, Levinthal suggested the existence of restricted folding ‘pathways’, which were later refined into the modern view of a funneled energy landscape (Dill & Chan, 1997 ▸; Onuchic & Wolynes, 2004 ▸). Many aspects of these landscapes remain poorly understood, including how proteins avoid kinetic traps (local minima) and the role that assisted folding (for example by chaperones) plays in shaping them.

A third question focuses on the practical problem of structure prediction and the design of algorithms that can predict the native state from the primary sequence of a protein given all available data, including the structures of related proteins or protein folds, homologous protein sequences and knowledge of polypeptide chemistry and geometry; such protein structure-prediction algorithms often involve physical principles, but recent work with machine-learning algorithms has shown that this need not necessarily be true (AlQuraishi, 2019*b* ▸). Moreover, solving the prediction task does not automatically advance our understanding of the folding process or address Levinthal’s paradox: it is most akin to a powerful and useful new experimental technique for structure determination.

Most machine-learning efforts, including *AlphaFold*2 (Jumper *et al.*, 2021 ▸), have focused on the structure-prediction problem (Gao *et al.*, 2020 ▸). Here too, there exist multiple subproblems and questions. Is the prediction to be based purely on a single protein sequence or can an ensemble of related sequences be used? Are structural templates permissible or is the prediction to be performed *ab initio*? And, perhaps most fundamentally, can it be demonstrated that there exists a single lowest energy conformation and that it has been successfully identified? The rationale for structure prediction is based on Anfinsen’s ‘thermodynamic hypothesis’, which states that in a physiological environment the lowest free-energy state of a protein is unique, and hence its corresponding 3D structure is also unique (Anfinsen, 1973 ▸). If this state is also in a sufficiently deep energetic well, then it is also rigid. Decades of experimental work support key aspects of this hypothesis for many proteins, whose crystallizability *ipso facto* demonstrates uniqueness and rigidity, at least under a given set of crystallization conditions. However, it is also the case that many proteins permit multiple crystallographic structures, and nuclear magnetic resonance (NMR) and cryo-electron microscopy (cryoEM) reveal a landscape of conformationally flexible proteins, including intrinsically disordered proteins (James & Tawfik, 2003 ▸). Moreover, some proteins, including those involved in viral membrane fusion, are not always found in their lowest energy states (White *et al.*, 2008 ▸). Improving our understanding of the reliability and applicability of protein structure prediction requires better characterization of the conditions under which any given protein can reach a unique conformation, if one exists. Current prediction methods, including *AlphaFold*2, do not explicitly model experimental conditions, yet they are trained on experimentally determined protein structures from the Protein Data Bank (PDB). Implicitly, then, these methods are best considered not as predictors of the lowest free-energy state under physiological conditions, but of the structured state under experimental conditions in which a protein is likely to crystallize (this is particularly true given the dominance of crystallographic structures in the PDB).

Multiple approaches have been taken to design algorithms for protein structure prediction. During the early decades of the field, the focus was on deciphering physically inspired maps that yield 3D structures from single protein sequences: the *Rosetta* modeling system was conceived within this paradigm (Rohl *et al.*, 2004 ▸). Starting in the early 2010s, with prevalent high-throughput sequencing and advances in statistical models of co-variation in the sequences of related proteins (Cocco *et al.*, 2018 ▸), the focus shifted from single-sequence models to models that exploit information contained in multiple sequence alignments (MSAs; Wang *et al.*, 2017 ▸). The approach taken by *AlphaFold*2 also operates on MSAs but leverages recent advances in neural networks to depart substantially from earlier models for protein structure prediction. In this review, we endeavor to explain (in Section 2) what attracted DeepMind to the protein structure-prediction problem. In Section 3, we provide a perspective on the *AlphaFold*2 CASP14 results relative to previous CASP experiments. In Section 4, we discuss the computational architecture used by *AlphaFold*2 and describe four related features that are central to its success: (i) the attention mechanism, which captures long-range dependencies in protein sequence and structure, (ii) the ensemble approach, specifically the use of MSAs as an input, (iii) the equivariance principle, which implements the idea of rotational and translational symmetry, and (iv) end-to-end differentiability, the glue that makes the entire approach work in a self-consistent and data-efficient manner. We conclude in Section 5 with a general discussion about the implications and limitations of *AlphaFold*2 and the interpretability of machine learning-based methods more broadly. Throughout our discussion, we turn to ideas from physics to ground our intuition.

## 2. From Go to proteins   

The machine-learning algorithms that are the specialty of DeepMind were first developed to tackle complex games such as Go and StarCraft2 (Silver *et al.*, 2016 ▸; Vinyals *et al.*, 2019 ▸). This is part of a long tradition of using games such as chess or Jeopardy to test new computational concepts. Three properties of Go and StarCraft2 made them amenable to machine-learning methods: the existence of a massive search space, a clear objective function (metric) for optimization and large amounts of data. Protein structure prediction shares some of these properties.

### 2.1. Massive search space   

The state-space complexity of Go (the number of attainable positions from the starting game configuration) is around 10^170^ (compared with ∼10^80^ atoms in the visible universe; van den Herik *et al.*, 2007 ▸). Prior to the introduction of *AlphaGo* in 2016 (Silver *et al.*, 2016 ▸), the consensus within the expert community was that a computer agent capable of winning a game of Go against a top-ranked professional player was at least a decade away. This was despite previous successes in chess, first achieved by the IBM Deep Blue system, which utilized brute-force computation. A brute-force approach to chess is possible in part due to its relatively modest state-space (van den Herik *et al.*, 2007 ▸), estimated at around 10^47^ configurations. To span the ∼10^123^-fold difference in complexity between the two games, *AlphaGo* used neural networks and reinforcement learning, marrying the pattern-recognition powers of the former with the efficient exploration strategies of the latter to develop game-playing agents with a more intuitive style of play than brute-force predecessors. However, proteins have an even larger theoretical ‘state-space’ than Go (Levinthal, 1968 ▸). Although DeepMind has yet to employ reinforcement learning in a publicly disclosed version of *AlphaFold*, its reliance on the pattern-recognition capabilities of neural networks was key to tackling the scale of protein conformation space. Helping matters was the fact that protein conformation space is not arbitrarily complex but is instead strongly constrained by biophysically realizable stable configurations, of which evolution has in turn explored only a subset. This still vast but more constrained conformation space represents the natural *umwelt* for machine learning.

### 2.2. Well defined objective function   

Games provide an ideal environment for training and assessing learning methods by virtue of having a clear winning score, which yields an unambiguous objective function. Protein structure prediction, unusually for many biological problems, has a similarly well defined objective function in terms of metrics that describe structural agreement between predicted and experimental structures. This led to the creation of a biennial competition (CASP) for assessing computational methods in a blind fashion. Multiple metrics of success are used in CASP, each with different tradeoffs, but in combination they provide a comprehensive assessment of prediction quality.

### 2.3. Large amounts of data   

*AlphaGo* was initially trained using recorded human games, but ultimately it achieved superhuman performance by learning from machine self-play (Silver *et al.*, 2016 ▸). Proteins, *prima facie*, present a different challenge from game playing, as despite the growing number of experimentally resolved structures there still exist only ∼175 000 entries in the PDB (Burley *et al.*, 2019 ▸), a proverbial drop in the bucket of conformation space. It is fortunate that these structures sufficiently cover fold space to train a program of *AlphaFold*2’s capabilities, but it does raise questions about the applicability of the *AlphaFold*2 approach to other polymers, most notably RNA. Furthermore, much of the success of *AlphaFold*2 rests on large amounts of genomic data, as the other essential inputs involve sequence alignments of homologous protein families (Gao *et al.*, 2020 ▸).

Finally, despite the similarities between protein structure prediction and Go, there exists a profound difference in the ultimate objectives. Whereas machine learning is optimized for predictive performance, analogous to winning a game, the protein-folding problem encompasses a broad class of fundamental scientific questions, including understanding the physical drivers of folding and deciphering folding dynamics to resolve Levinthal’s paradox (Dill & MacCallum, 2012 ▸).

## 3. *AlphaFold*2 at CASP14   

Many of the advances in structure prediction over the past two decades were first demonstrated in CASP experiments, which run every two years and focus on the prediction of protein structure. Typically, sequences of recently solved structures (not yet publicly released) or of structures in the process of being solved are presented to prediction groups with a three-week deadline for returning predictions (Kryshtafovych *et al.*, 2019 ▸). Two main categories exist: the more challenging ‘Free Modeling’ (FM) targets that have no detectable homology to known protein structures and require *ab initio* prediction, and ‘Template-Based Modeling’ (TBM) targets that have structural homologs in the PDB and emphasize predictions that refine or combine existing structures. Some TBM targets, often termed ‘Hard TBM’, exhibit minimal or no sequence homology to their structural templates, making the identification of relevant templates difficult. In all cases, CASP targets are chosen for their ability to stress the capabilities of modern prediction systems: a recently solved structure from the PDB chosen at random will on average be easier to predict than most CASP targets, including TBM targets.

CASP predictions are assessed using multiple metrics that quantify different aspects of structural quality, from global topology to hydrogen bonding. Foremost among these is the global distance test total score (GDT_TS; Kryshtafovych *et al.*, 2019 ▸), which roughly corresponds to the fraction of protein residues that are correctly predicted, ranging from 0 to 100, with 100 being a perfect prediction. Heuristically, a GDT_TS of 70 corresponds to a correct overall topology, 90 to correct atomistic details including side-chain conformations, and >95 to predictions within the accuracy of experimentally determined structures.

After a period of rapid progress in the first few CASP challenges, characterized by homology modeling and fragment assembly, progress in protein structure prediction slowed during the late 2000s and early 2010s. Much of the progress over the past decade has been driven by two ideas: the development of co-evolutionary methods (De Juan *et al.*, 2013 ▸) based on statistical physics (Cocco *et al.*, 2018 ▸) and the use of deep-learning techniques for structure prediction. Starting with CASP12 in 2016, both approaches began to show significant progress. CASP13 was a watershed moment, with multiple groups introducing high-performance deep-learning systems, and in particular the first *AlphaFold* achieving a median GDT_TS of 68.5 across all categories and 58.9 for the FM category (Senior *et al.*, 2020 ▸). These results were a substantial leap forward from the best median GDT_TS at CASP12 of ∼40 for the FM category (AlQuraishi, 2019*a* ▸).

**Figure 1:**
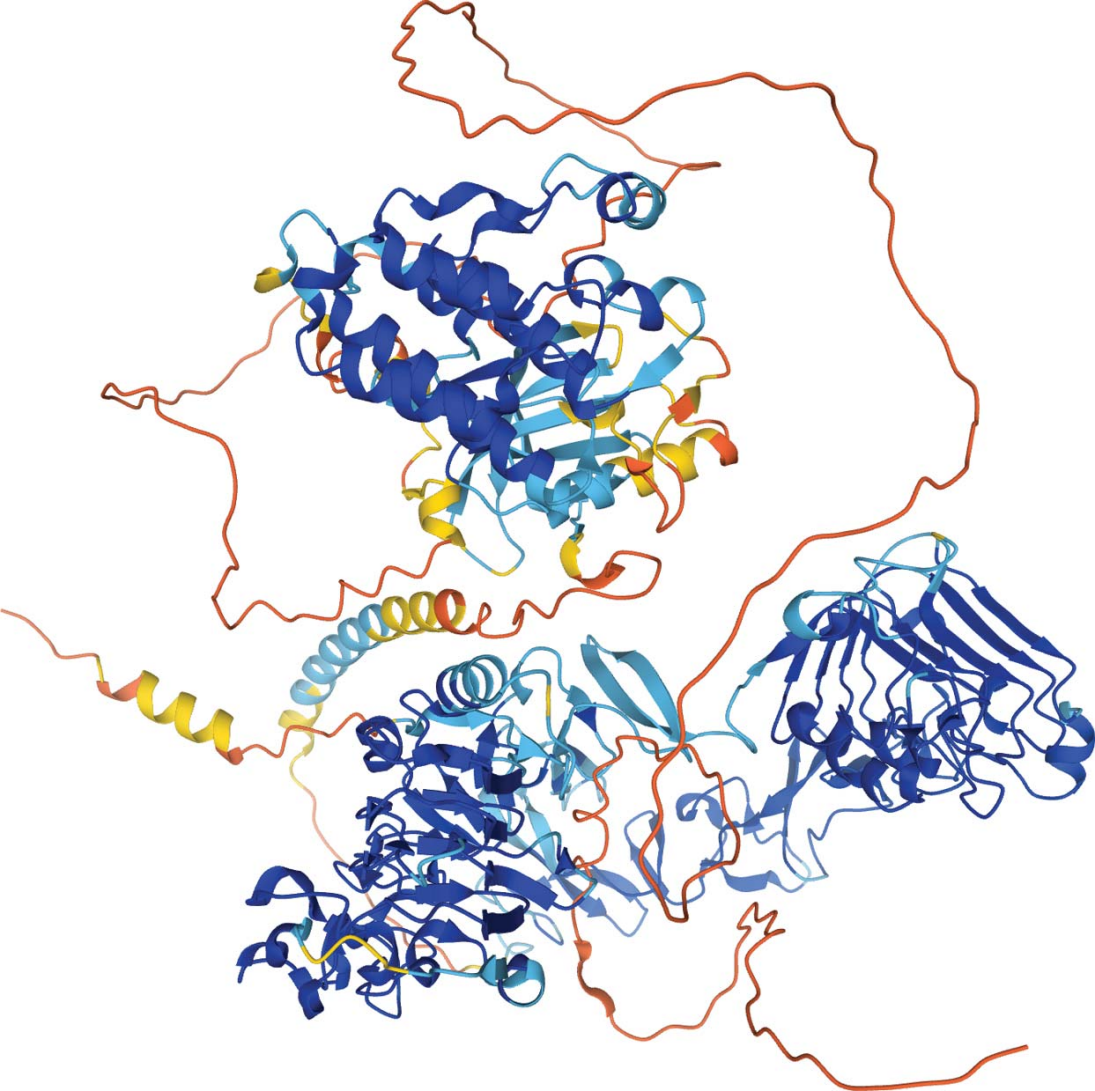
*AlphaFold*2 prediction of the full-length chain of human EGFR (UniProt ID: P00533) color coded by model confidence (dark blue, highly confident; dark orange, very low confidence). Individual domains are confidently predicted, but inter-domain arrangement is not, as evidenced by long unstructured linkers with very low model confidence.

In the most recent CASP14 experiment, *AlphaFold*2 achieved a median GDT_TS of 92.4 over all categories, a qualitative leap without historical parallel in CASP. *AlphaFold*2 correctly predicted atomistic details, including accurate side-chain conformations, for most targets, and achieved a median GDT_TS of 87 in the challenging FM category. When considering only backbone accuracy (*i.e.* the conformation of C^α^ atoms), *AlphaFold*2 achieves a root-mean-square deviation (r.m.s.d.) of <1 Å for 25% of the cases, <1.6 Å for half of the cases and <2.5 Å for 75% of the cases. When considering all side-chain atoms, *AlphaFold*2 achieves an r.m.s.d. of <1.5 Å for 25% of the cases, <2.1 Å for half of the cases and <3 Å for 75% of the cases. Despite the single-domain focus of CASP14, the publicly released version of *AlphaFold*2 appears capable of predicting structures of full-length proteins, although inter-domain arrangement remains a challenge (Fig. 1 ▸).

With respect to the ∼100 targets in CASP14, *AlphaFold*2 performed poorly on five, with a GDT_TS below 70. Three of these were components of oligomeric complexes, and two had structures determined by NMR. Poor performance on an oligomer may reflect the fact that *AlphaFold*2 was trained to predict individual protein structures (a different category exists for multimeric targets), reflecting the focus of CASP on predicting the structures of single-domain proteins. The poor performance of *AlphaFold*2 on NMR structures is more subtle to understand. On the one hand, it may be reflective of NMR structures being less accurate than those derived from X-ray crystallography. On the other hand, it may result from the dominance of crystallographic structures in the *AlphaFold*2 training data; in which case, *AlphaFold*2 is best understood as a predictor of structures under common crystallization conditions. Confirming this hypothesis and understanding its basis may enhance our understanding of experimentally determined structures.

*AlphaFold*2 is almost certain to impact experimental structural determination in other ways, for example by extending the applicability of molecular replacement as a means of tackling crystallographic phasing (McCoy *et al.*, 2021 ▸). As structural biology continues its shift towards protein complexes and macromolecular machines, particularly with the rapid growth in single-particle cryoEM, accurate *in silico* models of individual monomers may prove to be a valuable source of information on domains. Looking further ahead to *in situ* structural biology, *i.e.* the analyses of molecular structures in their cellular milieu, the field is expected to continue to evolve from determining the atomic details of individual proteins to the conformations of multi-component molecular machines, a task in which computational methods are playing increasingly important roles.

## 4. The *AlphaFold*2 architecture   

In models based on multiple sequence alignments (MSAs), up to and including the first version of *AlphaFold* (Senior *et al.*, 2020 ▸), summary statistics on inter-residue correlations were extracted from MSAs and used to model residue co-evolution (Cocco *et al.*, 2018 ▸). This serves as a source of information on spatial contacts, including contacts that are distant along the primary sequence and which play a critical role in determining 3D folds. The most advanced realizations of this approach employ residual neural networks, commonly used for image recognition, as pattern recognizers that transform the co-evolutionary signal in MSAs into ‘distograms’: matrices that encode the probability that any pair of residues will be found at a specific distance in space (Senior *et al.*, 2020 ▸). Inherently, such predictions are over-determined and self-inconsistent (too many distances are predicted, and they can be in disagreement with each other or not physically plausible) and physics-based engines are therefore necessary to resolve inconsistencies and generate realizable 3D structures (some exceptions exist; AlQuraishi, 2019*b* ▸; Ingraham, Riesselman *et al.*, 2019 ▸). The resulting fusion of statistical MSA-based approaches with machine-learning elements and classic physics-based methods represented a critical advance in structure prediction and paved the way for the deep-learning approaches used by *AlphaFold*.

*AlphaFold*2 departs from previous work on MSA-based structure prediction in several ways, firstly by starting with raw MSAs as inputs rather than summarized statistics, and secondly by predicting the final 3D structure rather than distograms as output (Jumper *et al.*, 2021 ▸). Two principal modules in *AlphaFold*2 were used to achieve this: (i) a neural network ‘trunk’ that uses attention mechanisms, described below, to iteratively generate a generalized version of a distogram and (ii) a structure module that converts this generalized distogram into an initial 3D structure and then uses attention to iteratively refine the structure and place side-chain atoms. The two modules are optimized jointly from one end to another (hence ‘end-to-end’ differentiable), an approach central to many of the recent successes in machine learning.

The attention mechanism is a key feature of the *AlphaFold*2 architecture. At its core, attention enables neural networks to guide information flow by explicitly choosing (and learning how to choose) which aspects of the input must interact with other aspects of the input. Attention mechanisms were first developed in the natural language-processing field (Cho *et al.*, 2014 ▸) to enable machine-translation systems to attend to the most relevant parts of a sentence at each stage of a translation task. Originally, attention was implemented as a component within architectures such as recurrent neural networks, but the most recent incarnation of the approach, so-called Transformers, have attention as the primary component of the learning system (Vaswani *et al.*, 2017 ▸). In a Transformer, every input token, for example a word in a sentence or a residue in a protein, can attend to every other input token. This is performed through the exchange of neural activation patterns, which typically comprise the intermediate outputs of neurons in a neural network. Three types of neural activation patterns are found in Transformers: keys, queries and values. In every layer of the network, each token generates a key–query–value triplet. Keys are meant to capture aspects of the semantic identity of the token, queries are meant to capture the types of tokens that the (sending) token cares about, and values are meant to capture the information that each token needs to transmit. None of these semantics are imposed on the network; they are merely intended usage patterns, and Transformers learn how best to implement key–query–value triplets based on training data. Once the keys, queries and values are generated, the query of each token is compared with the key of every other token to determine how much and what information, captured in the values, flows from one token to another. This process is then repeated across multiple layers to enable more complex information-flow patterns. Additionally, in most Transformers, each token generates multiple key–query–value triplets.

The *AlphaFold*2 trunk consists of two intertwined Transformers, one operating on the raw MSA, iteratively transforming it into abstract dependencies between residue positions and protein sequences, and another operating on homologous structural templates, iteratively transforming them into abstract dependencies between residue positions (*i.e.* a generalized distogram). If no structural templates are available, *AlphaFold*2 starts with a blank slate. The two Transformers are not independent, but update each other through specialized information channels. The structure module employs a different form of Transformer. Unlike the trunk Transformers, which are only designed to encode residue chain positions, the structure Transformer geo­metrically encodes residues in 3D space as a cloud of oriented reference frames, using a spatial attention mechanism that reasons over continuous Euclidean coordinates in a manner that respects the invariance of the shapes of proteins to rotations and translations. In the next sections, we discuss how these architectural features may explain the success of *AlphaFold*2 and point to future developments.

### 4.1. Inductive priors: locality versus long-range dependency   

To understand what motivated the use of Transformers in *AlphaFold*2, it is helpful to consider the types of inductive biases that neural network architectures impose and how they coincide with our understanding of protein biophysics. Some of the main difficulties in protein modeling stem from the substantial role that long-range dependencies play in folding, as amino acids far apart in the protein chain can often be close in the folded structure. Such long-range dependencies are ubiquitous in biology, appearing in transcriptional networks and networks of neurons, and, as suggested by some physicists, might be a consequence of evolutionary optimization for criticality (Mora & Bialek, 2011 ▸). In critical phenomena, correlation length diverges, *i.e.* events at different length scales make equally important contributions. For example, the distinction between liquid and gas phases disappears at criticality. This contrasts with most familiar situations in physics, where events on different spatial and temporal scales decouple. Much of our description of macroscopic systems is possible because large-scale phenomena are decoupled from the microscopic details: hydrodynamics accurately describes the motion of fluids without specifying the dynamics of every molecule in the fluid.

A major challenge in machine learning of proteins, and many other natural phenomena, is to develop architectures capable of capturing long-range dependencies. This too is a problem with roots in physics. The crux of the problem is that the study of long-range dependencies scales very poorly from a computational standpoint, as a system of *n* all-correlated particles necessitates *n* ^2^ computations just to capture pairwise (second-order) effects. One workaround involves the principle of locality, which is central to convolutional neural networks and much of 20th-century physics, particularly the formulation of field theories (Mehta *et al.*, 2019 ▸). Locality requires that events can only be directly influenced by their immediate neighborhood. For example, in Maxwell’s theory of electromagnetism, the interactions governing electromagnetic fields are local. Einstein’s theory of relativity enforces locality by imposing the speed of light as the maximal speed for signal propagation (and thus avoiding instantaneous action at a distance). In computer-vision tasks, features are often local, and convolutional networks naturally capture this relationship. Prior to *AlphaFold*2, almost all protein structure-prediction methods were based on convolutional networks, a carryover from treating contact maps and distograms as 2D images (Gao *et al.*, 2020 ▸). Despite their expediency, convolutional networks may be suboptimal for protein structure prediction due to their inability to capture long-range dependencies. Remedial solutions, such as the use of dilated convolutions in the first *AlphaFold* (Senior *et al.*, 2020 ▸), ameliorated the problem, but the fundamental limitation was not addressed.

Transformers, on the other hand, have a different inductive bias: all interactions within their receptive field, irrespective of distance, are *a priori* treated as equally important. Only after training do Transformers learn which length scales are the most relevant (Vaswani *et al.*, 2017 ▸). In its sequence-based representations (the Transformers in the trunk), *AlphaFold*2 does not alter this prior. For the structure module, *AlphaFold*2 is biased toward spatially local interactions, consistent with the nature of structure refinement, which primarily involves resolving steric clashes and improving the accuracy of secondary structures. Recent Transformer-based models of protein sequence other than *AlphaFold*2 have demonstrated substantial improvements in modeling sequence–function relationships, adding further evidence of the suitability of their inductive prior (Rives *et al.*, 2021 ▸; Rao *et al.*, 2021 ▸).

### 4.2. Sequence ensembles and evolution-based models   

The ensemble view of proteins, in which sets of homologous proteins are considered in lieu of individual sequences, emerged from the empirical observation that the precise amino-acid sequence of a protein is not always necessary to determine its 3D structure. Indeed, many amino-acid substitutions lead to proteins with similar structures, and the combinatorics of these substitutions suggest the plausibility of a function that maps sequence ensembles (families of homologous proteins) to single structures (Cocco *et al.*, 2018 ▸). One instantiation of this idea assumes that correlated mutations between residues reflect their physical interaction in 3D space. Inspired by methods from statistical physics (Potts models), this intuition can be formalized by describing a sequence ensemble with a probability distribution that matches the observed pairwise frequencies of coupled residue mutations while being maximally entropic (Cocco *et al.*, 2018 ▸; Mora & Bialek, 2011 ▸). In principle, the information contained in Potts models enables the reconstruction of protein structure by converting probabilistic pairwise couplings into coarse-grained binary contacts or finer-grained distance separations, which can then be fed as geometric constraints to a traditional folding engine. As discussed above, this was a key premise of many machine-learning methods prior to *AlphaFold*2 (Senior *et al.*, 2020 ▸).

*AlphaFold*2 re-examines the ensemble-based formulation by doing away with Potts models and other pre-defined statistical summaries. Instead, it relies on learnable primitives, specifically those captured by the Transformer, to directly extract information from raw pre-aligned sequence ensembles. This builds on prior work showing that Transformers can learn semantic representations of protein sequences (Rives *et al.*, 2021 ▸; Alley *et al.*, 2019 ▸) and reconstruct partially masked protein sequences, in effect inducing a Potts model of their own (Rao *et al.*, 2020 ▸). Transformer variants have also been introduced to handle more complex objects than simple sequential text, such as the inherently two-dimensional MSAs (Rao *et al.*, 2021 ▸; one dimension corresponds to alignment sequence length and the other to alignment depth).

*AlphaFold*2 remains an ensemble-based prediction method, predicting structures from families of related proteins instead of individual sequences. This may make it insensitive to sequence-specific structural changes that arise from mutations and suggests that it may not be effective when proteins have few homologues or are human-designed. This expectation comports with the behavior of co-evolution-based methods, and reflects the fact that such models do not learn a physical sequence-to-structure mapping function. However, *AlphaFold*2 did capture the general fold of Orf8 from SARS-CoV-2 with a GDT_TS of 87 based on only a few dozen sequences. Thus, it is possible that *AlphaFold*2 is capable of utilizing shallow MSAs.

### 4.3. Equivariance and the structure module   

Algorithms that reason over protein structure face the challenge that molecules do not have a unique orientation: the same protein rotated even slightly is an entirely different object computationally, despite being identical when in solution. *AlphaFold*2 accounts for this degeneracy by utilizing a 3D rotationally and translationally equivariant Transformer in its structure module, a construction rooted in symmetry principles from physics. This is known as an SE(3)-equivariant architecture (where SE stands for Special Euclidean).

Symmetries are central to physics because physical laws must obey them, and in many cases this imposes remarkably strong constraints on models. For example, the special theory of relativity put symmetry principles first, thereby dictating the allowed forms of dynamical equations. With the development of quantum mechanics, group theory, the mathematical framework accounting for symmetries, has played a central role, particularly in the development of the Standard Model of particle physics (Gross, 1996 ▸). Crystallographic point groups also capture the set of symmetries in crystals and play an essential role in experimental structure determination. Neural network architectures that model the natural world must also obey the symmetries of the phenomena that they are modeling (Bronstein *et al.*, 2021 ▸). Convolutional neural networks, commonly used for image-recognition tasks, obey the principle of translational equivariance; accordingly, translating an input in space before feeding it to a neural network is equivalent to feeding it unaltered and translating the output. The principle is yet more general and applies to intermediate network layers, establishing a commutative relationship between translations and neural network operations (Bronstein *et al.*, 2021 ▸). The difference between invariance (do not care) and equivariance (keep track) can be understood in terms of how proteins are represented to neural networks and how they are operated on. A matrix encoding all pairwise distances between protein atoms is an invariant representation because the absolute position and orientation of a protein in 3D space is lost; it therefore only permits invariant reasoning by a neural network. On the other hand, the raw 3D coordinates of a protein are neither invariant nor equivariant, but they permit a neural network to reason equivariantly because the absolute position and orientation of the protein are retained. Equivariance is actually achieved when the neural network generates (geometric) outputs that are translated and rotated in precisely the same way as the input representation of the protein.

Until recently, equivariance in neural networks was limited to translations in Euclidean space, but generalizing to molecular systems requires a more general approach to symmetries. Inspired by mathematical methods from physics, specifically group theory, representation theory and differential geometry, multiple machine-learning groups, starting in the mid-2010s and accelerating in the last two years, began generalizing neural network equivariance beyond translations and Euclidean spaces (Bronstein *et al.*, 2021 ▸; Cohen *et al.*, 2019 ▸). Initial efforts focused on discrete rotations and translations in two dimensions (for example 90° rotations; Cohen & Welling, 2016 ▸), which quickly advanced to continuous 2D transformations using harmonic functions (Worrall *et al.*, 2017 ▸). However, generalizing to three dimensions poses serious challenges, both computational and mathematical. Most early attempts reformulated the convolutional operations of neural networks as weighted mixtures of spherical harmonics (Thomas *et al.*, 2018 ▸; Weiler *et al.*, 2018 ▸), functions familiar from the mathematical formulation of the fast rotation function for molecular replacement (Crowther, 1972 ▸). Although elegant, these approaches are computationally expensive and may limit the expressivity of neural networks. Subsequent efforts have begun to diverge, with some aiming for greater expressivity at higher computational cost by pursuing group-theoretic constructions, particularly Lie algebras (Finzi *et al.*, 2020 ▸). Another subset has focused on computational efficiency and pursued graph-theoretic constructions, which are familiar to computer scientists, based on embedding equivariant geometrical information within the edges of graph neural networks or the query–key–value triplets of Transformers (Ingraham, Garg *et al.*, 2019 ▸; Satorras *et al.*, 2021 ▸). Outside of the *AlphaFold*2 structure module, no method involving thousands to tens of thousands of atoms (the scale of a protein) has as yet meaningfully leveraged equivariance.

The *AlphaFold*2 approach merges equivariance with attention using an SE(3)-equivariant Transformer. [Independently of *AlphaFold*2, a type of SE(3)-equivariant Transformer has been described in the literature (Fuchs *et al.*, 2020 ▸, 2021 ▸), but this construction is currently too computationally expensive for protein-scale tasks]. Unlike the trunk Transformer, which attends over residues along the protein chain in an abstract 1D coordinate system, the structure Transformer attends over residues in 3D space, accounting for their continuous coordinates in an equivariant manner. Structures are refined in Cartesian space through multiple iterations, updating the backbone, resolving steric clashes and placing side-chain atoms, all in an end-to-end differentiable manner (more on this property later). Unlike most previous methods, in which refinement is accomplished using physics-based methods, refinement in *AlphaFold*2 is entirely geometrical.

The ability to reason directly in 3D suggests that *AlphaFold*2 can extract patterns and dependencies between multiple entities and distinct scales of geometrical organization in protein structure, unlike 2D representations (for example distograms), which are inherently biased towards pairs of protein regions. Based on this, it stands to reason that SE(3)-equivariant Transformers may be useful for other problems in protein biology and molecular sciences, including quaternary complexes, protein–protein interactions and protein–ligand docking. The initial work that introduced the Transformer architecture appeared in 2017 (Vaswani *et al.*, 2017 ▸), with the first serious forays into SE(3)-equivariant architectures appearing in 2018 (Thomas *et al.*, 2018 ▸; Weiler *et al.*, 2018 ▸). Since then the field has flourished, with new conceptual innovations and improved computational implementations appearing at a rapid pace. Given this rapidity, it is reasonable to expect that better, faster and more efficient instantiations of *AlphaFold*2 and its generalizations are just around the corner.

### 4.4. End-to-end differentiability and the principle of unification   

In supervised machine learning, the aim is to learn a mathematical map from inputs to outputs (for example protein sequence to structure). Learning is achieved by changing the parameters of the map in a way that minimizes the deviations between the known ground truth, for example experimentally resolved structures, and predicted outputs. If the functions comprising the map are all differentiable (in the mathematical sense), optimization can be performed by iteratively evaluating the map and following its local gradient. This end-to-end differentiability condition greatly simplifies learning by enabling all parameters to be adjusted jointly instead of relying on a patchwork of disconnected steps, each of which is optimized independently (LeCun *et al.*, 2015 ▸).

Only a few machine-learning methods for protein structure prediction, including recent work by one of us (AlQuraishi, 2019*b* ▸), are end-to-end differentiable and therefore amenable to joint optimization. The paradigm for most existing methods is to take as input co-evolutionary maps derived from MSAs and predict inter-residue pairwise distances as output (the aforementioned distogram). The generation of 3D protein structure relies on a separate, non-machine-learned step. This approach is appealing as it reduces structure prediction to a simple 2D problem, both in input and output, and leverages machinery developed for computer-vision tasks. However, it prevents joint optimization of all components of the structure-prediction pipeline, and often results in self-inconsistent predictions. For example, predicted pairwise distances may not fulfill the triangle inequality. To resolve such inconsistencies, physics-based folding engines such as *Rosetta* incorporate predicted distances as soft constraints to generate the final 3D structure. *AlphaFold*2 changes this paradigm by being end-to-end differentiable, jointly optimizing all model components, including generation of the 3D structure, and thereby guaranteeing self-consistency. (The one exception is its use of MSAs, which are constructed outside of *AlphaFold*2 and used as inputs.)

Unlike symmetries such as SE(3) equivariance or allowable bond geometries, areas in which chemistry and physics offer useful prior knowledge and intuition, end-to-end differentiability is generally regarded as a computer-science concept distinct from physical principles. However, the capacity of end-to-end models to provide a unified mathematical formulation for optimization is reminiscent of the role that unification has played in the development of physics. The canonical example is Maxwell’s formulation of the fundamental equations of electromagnetism. Until Maxwell’s work, electricity, magnetism and light were considered to be separate phenomena with distinct mathematical descriptions. Their unification not only afforded a more accurate theory, but provided a unified mathematical framework in which the three phenomena interact with and constrain each other. By analogy, end-to-end differentiability allows different model components to constrain and interact with each other in a way that is convenient from a practical perspective and may reflect fundamental physical constraints in protein structure prediction. In the case of *AlphaFold*2, loss functions defined at the level of 3D structure propagate error through SE(3)-equivariant networks to refine the position of atoms in Cartesian space, which then further propagate error through 2D distogram-like objects implicitly encoding inter-residue distances. At each stage, different types of geometric information are used to represent the same object, and by virtue of end-to-end differentiability, they all constrain and reinforce one another, making the process more data-efficient. Crucially, these distinct stages make different computational and representational trade-offs; for example, distogram-like objects are by construction invariant to rotations and translations, making them computationally efficient, but are limited in their ability to represent the coordination of multiple atoms in distributed parts of the protein. On the other hand, the structure module in *AlphaFold*2 is explicitly engineered to behave equivariantly, but can readily represent interactions between multiple atoms distributed along the protein chain.

## 5. Interpretability in machine-learned protein models   

Reflecting on the crystallographic structure of myoglobin, the pioneers of structural biology conveyed a sense of disappointment at the first-ever protein structure; Kendrew and coworkers, commenting on their own discovery, proclaimed that

perhaps the most remarkable features of the molecule [myoglobin] are its complexity and its lack of symmetry. The arrangement seems to be almost totally lacking in the kind of regularities which one instinctively anticipates.

(Kendrew *et al.*, 1958 ▸). Prior to the broad availability of protein structures, it was anticipated that proteins would display simple and explicit regularities. Max Perutz attempted to build such simple models (Perutz, 1947 ▸), but Jacques Monod, an advocate of symmetry in biology, expected to find regularities in protein complexes rather than single proteins (a view that has proven to be largely correct; Monod, 1978 ▸). Physicists too expressed disappointment, with Richard Feynman declaring that

one of the great triumphs in recent times (since 1960), was at last to discover the exact spatial atomic arrangement of certain proteins … One of the sad aspects of this discovery is that we cannot see anything from the pattern; we do not understand why it works the way it does. Of course, that is the next problem to be attacked.

(Feynman *et al.*, 1964 ▸). Concerns about the ‘interpretability’ of protein structures therefore have a long history.

The success of machine learning in structure prediction again raises the question of whether it will be possible to obtain biophysical insight into the process of protein folding or whether prediction engines will simply serve as powerful black-box algorithms. The question of interpretability is not unique to structure prediction. Machine learning has also transformed natural language processing, yielding models such as GPT-3 that are capable of generating flowing prose and semantically coherent arguments (Brown *et al.*, 2020 ▸), but have yielded few improvements in our understanding of linguistic structures and their representation in the human mind. Will machine-learned protein models be any different?

The first and more pessimistic view posits that protein folding is inherently a complex system that is irreducible to general principles or insights by its very nature. In this view, it does not matter whether protein folds are inferred by machine learning or long-time-scale molecular-dynamics simulations capable of faithfully recapitulating folding pathways. Each fold is a unique result of innumerable interactions of all of the atoms in a protein, far too complex for formulation of the generalized abstraction that we commonly equate with ‘understanding.’ We note however that this view is already being challenged by useful heuristics that operate at the level of solved structures, such as describing folds in terms of recurrent motifs (for example α-helices and β-sheets) and our understanding of the role that hydrophobic interactions play in globular packing (Shakhnovich, 1997 ▸).

The second and more optimistic view posits that we will be able to interpret the emergent mathematical entities embodied in deep neural networks, including Transformers. While physical theories, unlike machine-learned models, greatly constrain the mathematical space of possible models, physicists must still associate mathematical entities with physical ones. A similar challenge exists in machine learning: we posit that it may be possible to translate machine perception to human perception and derive interpretable insights about sequence–structure associations and the nature of globular folds. Doing so would likely require better tools for probing the structure and parameterization of neural networks, perhaps permitting some degree of automation. Such efforts remain exceedingly rare in machine learning, but recent work on computer-vision systems has yielded promising early results (Cammarata *et al.*, 2020 ▸). Moreover, detailed and formal mathematical models for protein biophysics already exist (Brini *et al.*, 2020 ▸) and represent a natural framework for rigorous analyses of machine-learned models and structures. It is very likely that the future will incorporate aspects of both of the two extremes outlined above. Many fields of biomedicine will be advanced simply by having genome-scale structural information. Others, including protein design, may require deeper insight.

We end by drawing on historical parallels in physics. Quantum mechanics engendered heated debate that continues today (famously captured by the disputes between Einstein and Bohr) because it lacks an intuitive representation of the world despite its unprecedented empirical success. For example, the classical notion of a particle trajectory, which is so useful in most circumstances, simply does not make sense in the quantum realm (Laloë, 2001 ▸). To many physicists, quantum mechanics remains a mathematical formalism for predicting the probabilities that certain events can occur, and attempts to go beyond this into interpretations of reality are metaphysical distractions. This attitude is captured by David Mermin’s motto ‘shut up and calculate!’ (Mermin, 1989 ▸) and Stephen Hawking’s remark that ‘all I am concerned with is that the theory should predict the results of measurements’ (Hawking & Penrose, 2010 ▸). However, for some physicists, including Einstein, Schrödinger and most recently Penrose, it is necessary to replace, extend or re-interpret quantum mechanics to provide a satisfactory account of the physical world. No obvious consensus exists on what counts as ‘satisfactory’ in these efforts, but interpretability should not be dismissed as a purely philosophical concern since it often leads to the reformulation of fundamental scientific theories. Recall that Newton was criticized by contemporaries such as Leibniz for not providing a causal explanation for gravity, with its ‘action at a distance’, and Einstein, while working on special and general relativity, was deeply influenced by Leibniz’s and Mach’s criticism of Newtonian mechanics. He specifically sought to put gravity on a more solid physical foundation by avoiding action at a distance. How we perceive the role and value of machine learning depends on our expectations. For Hawking, predictive power might be all that we need; for Einstein, new mathematical and conceptual tools may yield new understanding from neural networks. Physical understanding may also take time to develop. In the case of quantum mechanics, John Bell revisited the Bohr–Einstein debate almost forty years later, establishing the inequalities that bear his name and distinguish between classical and quantum behaviors (Bell, 2004 ▸; Aspect, 2016 ▸). This insight helped to enable many subsequent technological advances, including quantum computers (Nielsen & Chuang, 2010 ▸). Future versions of such quantum computers, with their ability to simulate quantum-chemical systems, may in turn shed light on the protein-folding problem.
